# Forest Attributes and Soil Moisture Availability Drive Ecosystem Multifunctionality of Forests in Eastern Tibetan Plateau, China

**DOI:** 10.3390/plants15030518

**Published:** 2026-02-06

**Authors:** Ming Ni, Peng Luo, Hao Yang, Honglin Li, Yue Cheng, Yu Huang

**Affiliations:** 1 CAS Key Laboratory of Mountain Ecological Restoration and Bioresource Utilization & Ecological Restoration and Biodiversity Conservation Key Laboratory of Sichuan Province, Chengdu Institute of Biology, Chinese Academy of Sciences, Chengdu 610041, China; niming22@mails.ucas.ac.cn (M.N.); lihl@cib.ac.cn (H.L.); huangyu21@mails.ucas.ac.cn (Y.H.); 2 University of Chinese Academy of Sciences, Beijing 101400, China; 3 Department of Biology, Aarhus University, 8000 Aarhus, Denmark; yuecheng@bio.au.dk

**Keywords:** ecosystem multifunctionality, biodiversity, functional traits, stand structure, Qinghai-Tibetan Plateau

## Abstract

Forests deliver multiple essential ecosystem functions, and most natural forests occur in highly heterogeneous environments and span different developmental stages. Despite this complexity, the relative influences of biotic and environmental drivers on ecosystem multifunctionality (EMF) remain insufficiently understood across temporal and spatial scales. Here, we surveyed forests along elevational (1800–3500 m) and successional (early to late) gradients on the eastern Tibetan Plateau, quantify how climate, soil properties, and forest attributes (diversity, stand structure, and functional traits) regulate EMF. EMF was constructed from eight indicators representing nutrient cycling, plant productivity, and water conservation. Further, we assessed variation in biodiversity effects, including selection and complementarity effects. We found that soil moisture, functional diversity, and the coefficient of variation in stand diameter exert significant positive effects on EMF, whereas species richness—the most commonly used diversity metric—shows no significant effect. Mean annual temperature and soil bulk density, by contrast, have significant negative effects. The strengths of both selection and complementarity effects vary along elevational and successional gradients, with complementarity effects becoming markedly stronger at higher elevations. Overall, our findings reveal the mechanisms through which climate, soil properties, and forest attributes jointly regulate EMF, underscoring the pivotal roles of plant functional diversity and structural heterogeneity in sustaining the multifunctionality of subalpine forests. Results provide a robust empirical foundation for improving natural forest EMF and restoration management.

## 1. Introduction

The relationship between biodiversity and ecosystem functioning (BEF) has long been at the forefront of ecological research, not only central to understanding the mechanisms underlying natural ecosystem processes but also theoretically crucial for addressing global risks such as climate change and biodiversity loss [1,2,3,4]. Forests, which cover about 31% of the global land surface [5], provide essential ecosystem functions such as timber production, soil and water conservation, and nutrient cycling, etc. [6,7,8]. Forest ecosystems typically support multiple functions simultaneously, which may involve synergies or trade-offs. This ability to maintain multiple functions and their interactions is captured by the concept of ecosystem multifunctionality (EMF) [9,10,11]. Consequently, BEF relationships assessed from a multifunctional perspective may differ markedly from those derived from single-function analyses. However, most previous studies have focused on individual ecosystem functions, one-sided observation limiting our ability to capture the complexity of ecosystem [12,13,14]. Although biodiversity is widely viewed as a key driver of forest EMF, the strength and direction of its effects remain inconsistent, particularly in natural forests with complex structures and diverse histories [3,15,16]. BEF relationships are shaped by factors such as climate [17], vegetation type [18], developmental or successional stage [19,20], regeneration mode [15], and the choice of diversity metrics [4], and that the resulting associations are predominantly positive [6,7] but can also be neutral [2,21] or negative [22]. Understanding the relative importance and of these regulatory factors is therefore a central focus of current research.

Two primary hypotheses explain BEF effects currently [1]. Complementarity effects are reflected in niche differentiation and functional synergy among species, whereby different species “specialize” in resource use, reducing waste and enhancing overall ecosystem functioning. Complementarity can be quantified using functional diversity indices [23,24]. In contrast, the core of the selection effect lies in the “dominance” of the most functionally or competitively superior species in the ecosystem, whose presence or absence directly determines the overall ecosystem functioning, with their contribution far exceeding that of other species. Selection effects are typically represented by the community-weighted mean (CWM) of plant trait values [25]. In natural forests, these mechanisms are not mutually exclusive but often act synergistically to decide BEF relationships [26,27]. Crucially, their relative importance is context-dependent. For example, selection effects dominate in temperate forests where productivity is tightly linked to traits like maximum height [20,22], whereas complementarity effects become more prominent in species-rich subtropical forests, where functional diversity is a stronger predictor [28]. However, across environmental or successional gradients, evidence remains limited on how the relative importance of selection versus complementarity effects shifts.

Biodiversity does not always directly indicate the extent of plant niche differentiation or resource-use efficiency, since different species can occupy similar positions within the stand’s vertical structure [29,30]. By contrast, structural diversity constitutes a key potential driver of forest ecosystem functioning [31]. It generates multidimensional niches through vertical stratification (canopy and understory) and horizontal spatial heterogeneity, promoting species coexistence and niche differentiation in resource use [32,33]. Complex canopy architecture enhances photosynthetic efficiency, while trees varying in height and DBH reduce direct competition for light [34,35]. Moreover, ecosystem functioning depends on both biotic factors and abiotic environmental variables [25,36,37]. When environmental factors such as climate variation and soil physicochemical properties are simultaneously considered, does biodiversity and other forest attributes still exert a prominent influence on ecosystem functioning? These questions obviously limit our comprehensive understanding of the mechanisms underlying BEF relationships.

To our knowledge, despite the fact that forests particularly in biodiversity-rich regions, occur in highly heterogeneous environments and undergo distinct developmental stages [38], few studies have simultaneously examined BEF relationships across both environmental gradients and successional trajectories [16]. The mountainous regions of southwestern China constitute one of the world’s 36 biodiversity hotspots, where pronounced climatic differentiation along elevation has produced a complete spectrum of vegetation zones [39,40]. Historical logging, disturbance have created extensive areas of secondary natural forests at different stages of recovery, offering an exceptional natural laboratory for studying BEF relationships [41,42]. Capitalizing on this unique setting, our study aims to provide an integrated understanding of how BEF relationships and their underlying mechanisms shift across both spatial and temporal gradients. To achieve this, we integrate forest survey data from the Gongga Mountains (representing a pronounced elevational/climatic gradient) and the Miyaluo forest region (representing a forest successional gradient). Specifically, we address the following three key questions: (i) What is the relationship between biodiversity and EMF in subalpine forests? (ii) To what extent do biodiversity, environmental factors, and structural diversity regulate ecosystem multifunctionality in subalpine forests? (iii) How do biodiversity effects (selection and complementarity effect) vary across spatial and temporal gradients?

## 2. Results

### 2.1. The Changes in EMF in Spatiotemporal Dynamics

**Figure 1:**
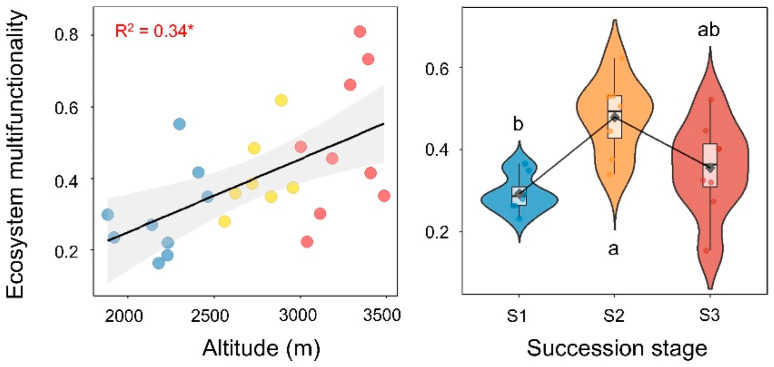
Spatiotemporal dynamics of ecosystem multifunctionality (EMF) in subalpine forests. Along the elevational gradient on the eastern slope of Mount Gongga, blue, yellow, and red points indicate low (1800–2400 m), mid (2400–2900 m), and high (2900–3500 m) elevation bands, respectively. Successional stages from early to late succession were classified into the following three categories: broadleaf forest (S1), mixed conifer–broadleaf forest (S2), and conifer forest (S3). * *p* < 0.05, and shaded area denotes the 95% confidence intervals of the fitted model. Different lowercase letters denote significant differences (*p* < 0.05).

As shown in Figure 1, EMF increased gradually with elevation (R^2^ = 0.34, *p* < 0.05). Along the successional gradient, EMF peaked at the mid-successional stage (*p* < 0.05) but did not differ significantly from the late-successional stage.

### 2.2. The Relationship Between EMF and Biotic/Environmental Factors

**Figure 2:**
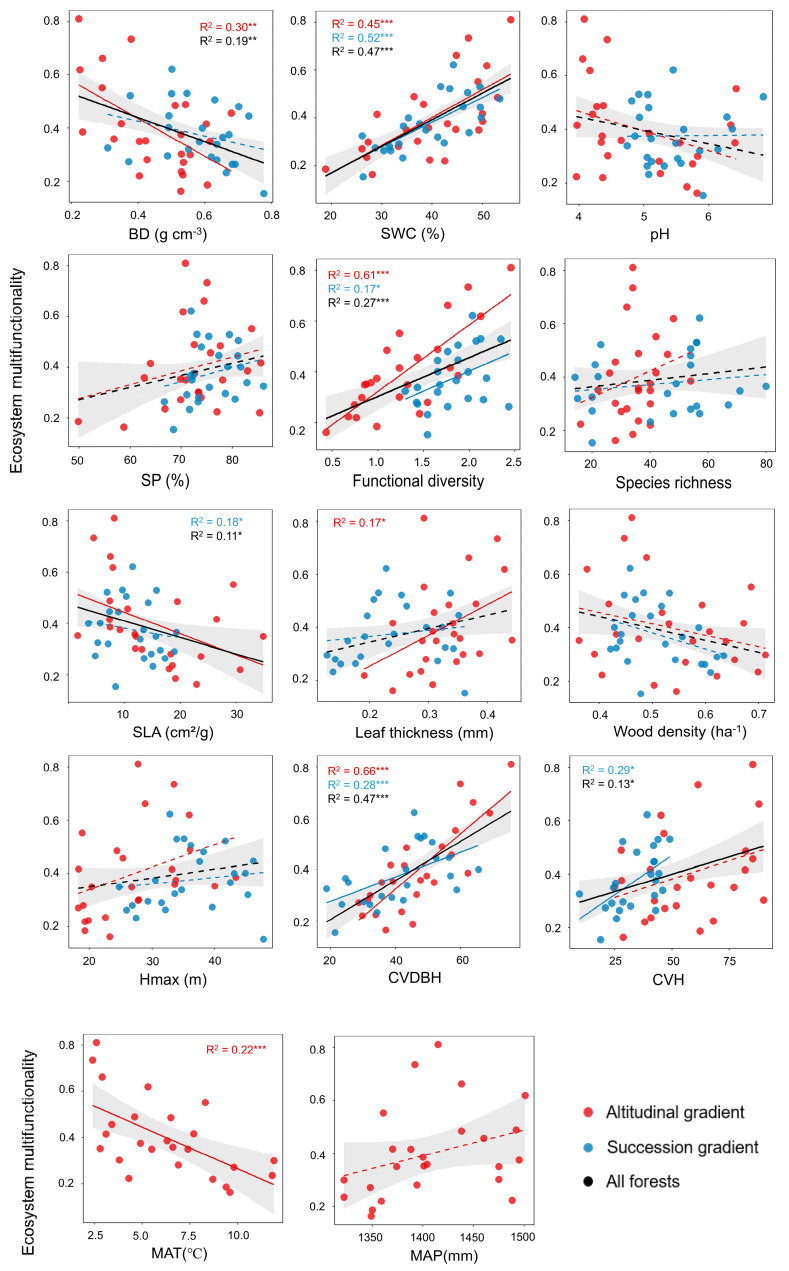
Relationships between ecosystem multifunctionality (EMF) and explanatory variables. Explanatory variables included soil bulk density (BD), soil water content (SWC), pH, soil porosity (SP), functional diversity (Fdis), species richness (SR), specific leaf area (SLA), leaf thickness, wood density, community maximum tree height (Hmax), coefficients of variation in DBH (CVDBH) and tree height (CVH), mean annual temperature (MAT), and mean annual precipitation (MAP). Black lines represent all forests, red lines represent forests along the elevational gradient, and blue lines represent forests along the successional gradient. Solid and dashed lines indicate significant and non-significant relationships, respectively. Significance levels are indicated as * *p* < 0.05, ** *p* < 0.01, and *** *p* < 0.001. Shaded area denotes the 95% confidence intervals of the fitted model.

Regarding soil properties, soil bulk density was negatively correlated with EMF (R^2^ = 0.19, *p* < 0.01), whereas soil water content promoted and explained a substantial proportion of EMF variation (R^2^ = 0.47, *p* < 0.001) (Figure 2). Soil pH and soil porosity showed no significant correlations. In terms of biodiversity, functional diversity was strongly positively correlated with EMF (R^2^ = 0.27, *p* < 0.001), while species richness showed no apparent correlation. Among community-level functional traits, specific leaf area (SLA) was positively correlated with EMF (R^2^ = 0.11, *p* < 0.01). All stand structural diversity metrics—coefficients of variation in DBH and tree height—were significantly positively correlated with EMF (R^2^ = 0.47, *p* < 0.01; R^2^ = 0.13, *p* < 0.05). Among climatic factors, EMF decreased with increasing mean annual temperature (R^2^ = 0.22, *p* < 0.001).

### 2.3. Relative Influence of Biotic and Environmental Factors on EMF

**Figure 3:**
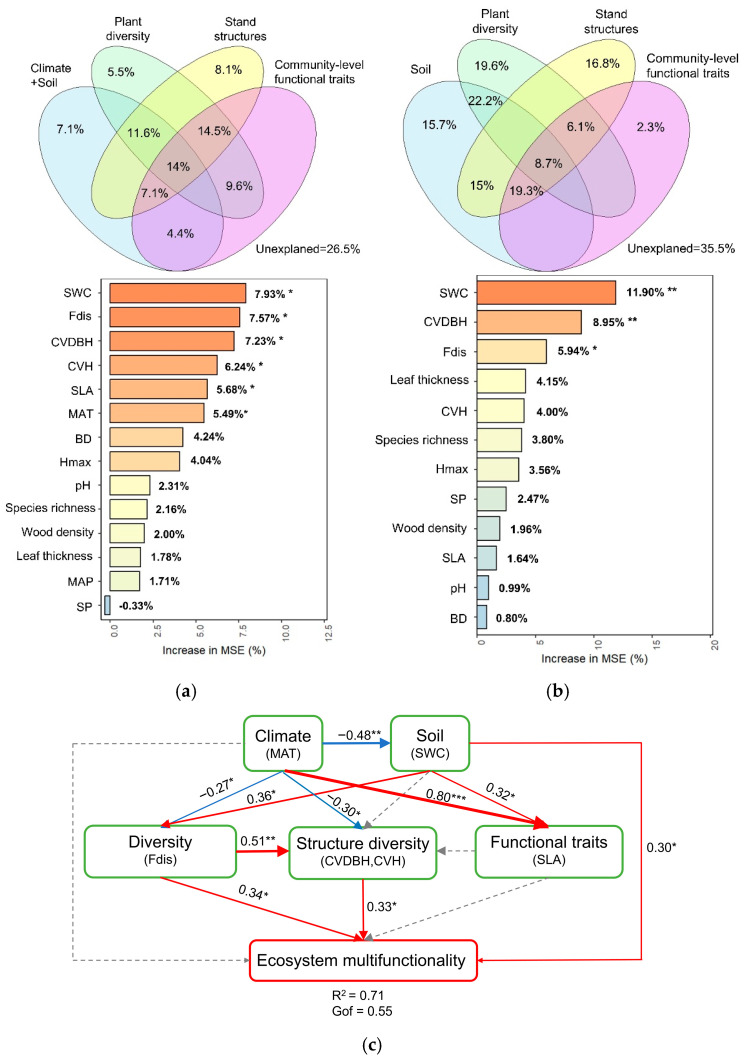
Relative contributions of biotic and environmental factors to ecosystem multifunctionality (EMF) in subalpine forests. (**a**) Elevational gradient (**b**) successional gradient. Venn diagrams show the relative contributions of climate, soil, plant diversity, stand structure, and community-level functional traits to variation in EMF. (**c**) Partial least squares path modeling (PLS-PM) identify the key explanatory variables, including soil bulk density (BD), soil water content (SWC), pH, soil porosity (SP), functional diversity (Fdis), species richness (SR), specific leaf area (SLA), leaf thickness, wood density, community maximum tree height (Hmax), coefficients of variation in DBH (CVDBH) and tree height (CVH), mean annual temperature (MAT), and mean annual precipitation (MAP). Note that PLS-PM analysis across the succession gradient was not performed due to an insufficient number of key explanatory variables. Red and blue arrows indicate significant positive and negative relationships, respectively. Numbers on the lines are standardized path coefficients. Significance levels are indicated as * *p* < 0.05, ** *p* < 0.01, and *** *p* < 0.001.

Variance partitioning analysis revealed that environmental factors (climate and soil), plant diversity, stand structure, and community functional traits collectively explained 73.5% of the variation in EMF along the elevational gradient and 64.5% along the successional gradient (Figure 3a,b). Individually, the proportions of EMF variation explained were: stand structure (8.1%, 16.8%), plant diversity (5.5%, 19.6%), and environmental factors (7.1%, 15.7%). The PLS-PM analysis revealed that plant diversity exerts a strong indirect positive effect on EMF by promoting stand structural diversity (Figure 3c). These findings were corroborated by partial correlation analysis (Figures S4 and S5).

### 2.4. Dynamics of Biodiversity Effects

**Figure 4:**
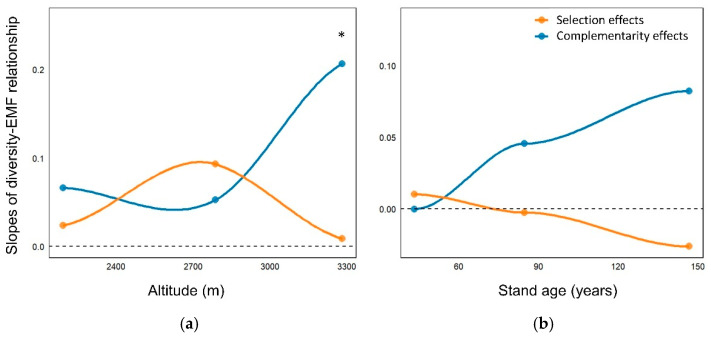
Complementarity (Fdis) and selection (CWM) effects on ecosystem multifunctionality (EMF) in subalpine forests. (**a**) Elevational gradient (**b**) successional gradient. Effect sizes represent the slopes of linear models relating Fdis and CWM to EMF. Curves show generalized additive model fits along the elevational and successional (stand-age) gradients. Significance levels are indicated as * *p* < 0.05.

Along the elevational gradient, the selection effect increased to a peak at approximately 2700 m before declining at higher elevations. Across the successional gradient, it declined progressively, reaching its lowest level in the late-successional stage (Figure 4a,b). In contrast, the complementarity effect remained relatively stable from low to mid elevations but increased sharply at higher elevations, and it likewise rose steadily across succession. Notably, complementarity effects were significantly stronger than selection effects at high elevations and in late-successional coniferous forests (*p* < 0.05), whereas the two did not differ significantly in other contexts (*p* > 0.05).

## 3. Discussion

### 3.1. Effects of Biotic Factors on EMF

Species richness has been shown to positively influence ecosystem multifunctionality (EMF) in grasslands [18], wetlands [43], alpine ecosystem [23] and certain types of forests [12,25,28]. Nevertheless, in subalpine forests, species richness did not significantly affect EMF along either the elevational or successional gradients, although weak positive trends were observed. This pattern may reflect the constrained range of species richness in high-elevation areas or mid-to-late successional stages, where conifer species dominate the community and only a few broadleaf species, such as birch, rhododendron, and oak, are present [41,44]. This limited species pool, coupled with potential high functional redundancy among the dominant conifers, may buffer changes in species number, directing the primary drivers of EMF towards functional attributes rather than taxonomic counts [14,28]. In the meanwhile, EMF levels are often relatively high, leading to a non-significant relationship between species richness and EMF. By contrast, communities with higher functional diversity exhibited higher EMF, which can be further illustrated by the role of complementarity effects [45]. When species differ substantially in their functional traits, their ecological niches tend to be complementary and interspersed, reducing direct interspecific competition and enhancing resource use efficiency [46,47]. This promotes multiple ecosystem processes, including biomass accumulation and nutrient cycling, thereby increasing overall ecosystem multifunctionality [48,49]. Community-level functional traits, such as specific leaf area (SLA) and leaf thickness, were also significantly correlated with EMF, indicating that selection effects contribute to EMF regulation. Previous studies on BEF relationship have emphasized the joint roles of selection and complementarity effects in shaping EMF [50,51,52,53]. However, we find that the intensity of these effects is not constant. In subalpine forests, the relative strength of these effects varied along elevational and successional gradients. According to the stress-gradient hypothesis [54], facilitative and mutualistic interactions among species become more prominent under increasing environmental stress, while intense competition is reduced [55,56]. Consistent with this hypothesis, complementarity effects were significantly stronger than selection effects at high elevations in subalpine forest, supporting the view that BEF relationships are amplified under harsh climatic conditions and may weaken—or even become negative—under more favorable environments [1].

Forest structural diversity independently explained a substantial portion of EMF variation in our variance partitioning analysis, and the coefficient of variation in DBH was significant in both linear and PLS-PM models, highlighting structural diversity as a key regulator of EMF in subalpine forests. Vegetation physical structure is closely linked to ecosystem functioning; by enhancing resource and space use efficiency—particularly light capture—structural diversity promotes niche differentiation and reduces direct competition among species [57,58]. Specifically, complex vertical and horizontal structures create heterogeneous microhabitats and resource gradients (e.g., light, humidity), enabling the coexistence of species with varying ecological strategies [39,59]. Across diverse region ecosystems, including North American [31,60], European [61], and Asian forests [28,62], structural diversity has consistently emerged as a strong predictor of ecosystem functioning, often outperforming traditional metrics such as plant species richness. Consequently, management practices should place greater emphasis on localized interpretation and application of BEF relationships, moving beyond the simplistic adoption of plant diversity as a primary or sole criterion [31,63]. This shift necessitates a deeper mechanistic understanding of how biodiversity influences ecosystem processes, even though it challenges prevailing assumptions that often report universally positive effects [34,64,65]. Structural diversity, being a readily quantifiable forest attribute, may offer novel insights and practical approaches for guiding effective forest management and restoration. Harnessing this attribute could potentially enhance both individual functions and overall multifunctionality in forest ecosystems.

### 3.2. Effects of Environmental Factors on EMF

Soil bulk density, a key indicator of soil compaction, directly constrains the physical environment for organism survival and activity when elevated [66,67]. High bulk density reduces soil porosity and aeration, mechanically hindering root penetration and limiting access to deep water and nutrients [68,69,70]. In addition, hypoxic conditions strongly suppress the activity of aerobic microbes, including decomposers and nitrifying bacteria, thereby impeding critical belowground processes such as organic matter decomposition and nutrient mineralization [71,72,73]. Consequently, elevated soil bulk density restricts the potential for high ecosystem functioning in subalpine forests. Soil water content is closely linked to plant growth and development [74]. Adequate water alleviates drought stress, promotes photosynthesis and biomass accumulation, and facilitates metabolic cycling and litter production [75,76]. Favorable soil conditions—characterized by high water content and low bulk density—often co-occur to support richer plant communities, enabling ecosystems to perform carbon sequestration, nutrient cycling, and other processes more efficiently and simultaneously [43,77]. In this context, soil bulk density and water content act as two complementary regulators of EMF as follows: the former establishes a “physical ceiling” for ecosystem function, whereas the latter provides a “physiological driving force.”

In subalpine forests along the elevational gradient, mean annual temperature (MAT) was significantly negatively correlated with ecosystem multifunctionality (EMF), indicating that EMF increased as temperature decreased with elevation. This pattern contrasts with observations from many energy-limited ecosystems at large spatial scales [6,78], highlighting the unique ecological processes and functional trade-offs in subalpine systems. As elevation rises and temperature declines, environmental filtering favors more conservative functional strategies [79,80,81]. High-elevation conifers allocate resources toward nutrient retention and the maintenance of defensive structures, often attaining biomass far exceeding that of co-occurring broadleaf species [82,83]. Additionally, low temperatures strongly suppress microbial decomposition, reducing the short-term rate of nutrient cycling while promoting the accumulation of soil organic matter and litter carbon stocks [84,85]. This shift toward a slower, more retentive nutrient economy and enhanced carbon storage that supports high EMF in these cold environments, prioritizing long-term stability over rapid turnover.

## 4. Materials and Methods

### 4.1. Study Area

**Figure 5:**
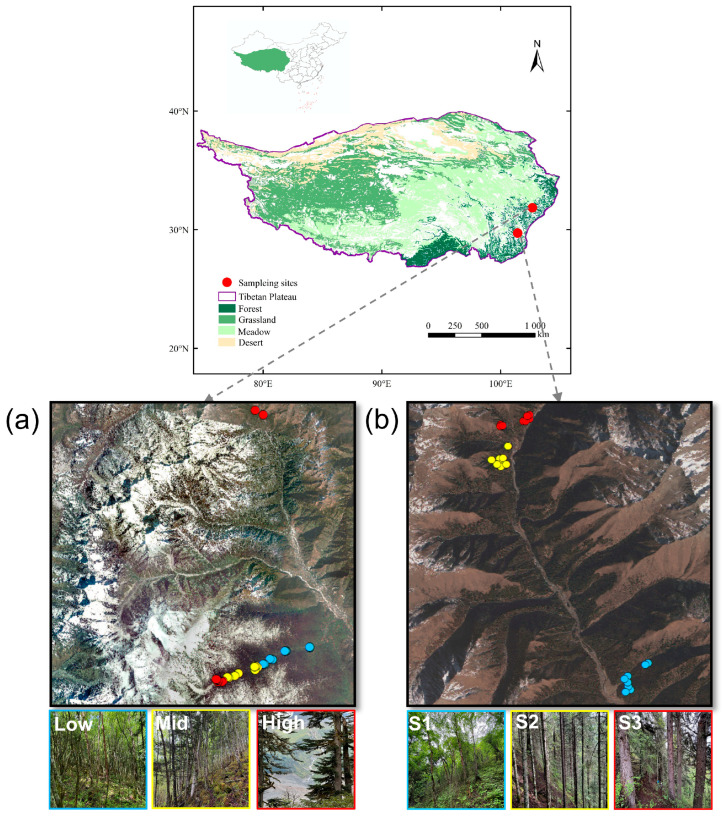
Study area. (**a**) Elevational gradient along the eastern slope of Mount Gongga, classified from bottom to top as low (1800–2400 m), mid (2400–2900 m), and high (2900–3500 m). (**b**) Successional stages from early to late, divided into three categories: broadleaf forest (S1), mixed conifer–broadleaf forest (S2), and conifer forest (S3). The blue, yellow, and red dots correspond to the “Low,” “Mid,” and “High” quadrats and the “S1,” “S2,” and “S3” quadrats, respectively.

The elevational gradient experiment was carried out on the eastern slope of Mount Gongga (101°30′–102°15′ E, 29°20′–30°20′ N), where the climate is dominated by the Asian monsoon. The region has a mean annual temperature of 4.3 °C and mean annual precipitation of 1900 mm, with most rainfall occurring from June to December [86]. Along this slope, temperature declines by 0.67 °C and precipitation increases by 67.5 mm per 100 m of elevation gain, accompanied by corresponding shifts in soil properties and vegetation types across vertical climatic zones. Soils are mainly derived from glacial debris and colluvium produced through the weathering of Permian quartz schist and Cenozoic feldspathic granite. Sampling plots were established between 1800 and 3500 m a.s.l. and grouped into the following three elevational bands: low (1800–2400 m), mid (2400–2900 m), and high (2900–3500 m), corresponding to broadleaf forests, mixed conifer–broadleaf forests, and subalpine coniferous forests with shrubland interzone, respectively [87] (Figure 5a).

The successional gradient experiment was conducted in Miyaluo Town, Lixian County, Sichuan Province (31°24′–31°55′ N, 102°35′–103°40′ E), at elevations of 3000–3300 m. The region has a monsoonal montane climate with cold winters and cool summers, and a mean annual temperature of approximately 2.1 °C [42]. Mean annual precipitation is about 864 mm, with 30% falling between October and April. Soils are predominantly brown forest soils rich in organic matter and showing clear vertical stratification. Using a space-for-time substitution, successional stages were classified into three categories primarily based on species composition and community structure, and further validated by stand age. The stages are: broadleaf forest (birch basal area > 70%; average stand age ~40–50 years), mixed conifer–broadleaf forest (conifer basal area 30–70%; average stand age ~80–90 years), and conifer forest (fir and spruce basal area > 80%; average stand age ~140–150 years). These were designated as stages S1 to S3, respectively (Figure 5b). The stand ages, obtained from local forestry records, confirm that the identified stages represent a clear temporal sequence of post-logging forest recovery.

Both the elevational (Gongga) and successional (Miyaluo) gradients are situated within the same biogeographic region (Hengduan Mountains) and ecosystem type (subalpine forest), ensuring their comparability within the framework of this study.

### 4.2. Community Survey

From June to September 2024, during the peak growing season of the subalpine forests, we conducted community surveys along both elevational and successional gradients. Along Mount Gongga, three survey plots were established within each 200 m elevational interval, totaling 25 plots. In the Miyaluo forest region, 24 plots were selected along the forest successional gradient, corresponding to three successional stages with eight plots per stage. Plots were systematically established in areas that had undergone natural regeneration with minimal human disturbance or grazing. Plot sizes were 1 × 1 m for herbaceous vegetation, 5 × 5 m for shrubs, and 20 × 20 m for trees. To maintain spatial independence and avoid pseudo replication, the distance between plots was kept at least 300 m. All plant species were identified to the species level with the assistance of local botanists, and species nomenclature in the database was standardized according to the Flora of China (http://www.efloras.org). Tree diameter at breast height (DBH) was measured at 1.3 m using a diameter tape.

### 4.3. Biotic Factors Measurements

In this study, taxonomic diversity was quantified as plant species richness. Functional diversity (functional dispersion) was calculated using the following four traits closely linked to tree growth and development: maximum tree height, specific leaf area, leaf thickness, and wood density. Community-level trait values were weighted by relative basal area, defined as the percentage of the total basal area contributed by each species. Maximum tree height was measured in the field for each species, whereas wood density values were obtained from a wood density database [88]. Following globally standardized protocols for plant functional trait measurement [89,90], at least three mature individuals per species were selected. From each individual, ten fully expanded, healthy leaves were randomly sampled to measure leaf thickness, leaf area, and specific leaf area. When sampling was not feasible due to low biomass or species rarity, corresponding trait data were retrieved from the TRY database (www.try-db.org). Forest structural diversity was quantified using the coefficients of variation in diameter at breast height and tree height [32].

### 4.4. Environmental Factors Measurements

Mean annual precipitation and temperature along the elevational gradient were obtained from the WorldClim database (http://www.worldclim.org) at a spatial resolution of 30 arc seconds [6]. Soil sampling was conducted using a five-point method, collecting surface soil from 0 to 20 cm depth. At each sampling point, five soil samples were combined into a composite sample for physicochemical analysis. Soil pH was measured using a glass electrode meter (InsMark™ IS126, Shanghai, China) in a 1:2.5 soil-to-water mixture. Soil organic carbon (SOC) content was determined by the potassium dichromate oxidation method, total nitrogen (TN) was analyzed by the Kjeldahl method, and available nitrogen was measured using the Kjeldahl nitrogen method. Total phosphorus (TP) and available phosphorus (AP) were determined using the molybdenum-antimony colorimetric method. Three intact soil cores (100 cm^3^ each) were collected using cutting rings for determination of soil physical properties. Soil bulk density (BD) was calculated as the oven-dried (105 °C for 24 h) soil mass divided by the core volume, following standard protocols. Soil water content (SWC) was measured gravimetrically by weighing fresh soil before and after oven drying at 105 °C to constant weight. Maximum water-holding capacity (MWHC) was assessed by saturating the cores under capillary action for 24 h, followed by gravitational drainage for 24 h; the retained water content was then measured and expressed as a percentage of dry soil weight. Soil porosity was measured by gas displacement using helium after vacuum degassing (10^−3^ bar, 1 h).

### 4.5. Ecosystem Multifunctionality

In this study, the following eight parameters quantifying key ecosystem functions were selected to assess ecosystem multifunctionality (EMF): aboveground biomass, root biomass, soil water-holding capacity, soil organic carbon, total soil nitrogen, available nitrogen (ammonium and nitrate), total soil phosphorus, and available phosphorus. These parameters serve as quantifiable proxies for fundamental ecosystem processes [24,50], representing the following four critical areas: primary productivity (biomass), soil water regulation (water-holding capacity), carbon sequestration (soil organic carbon), and nutrient cycling and storage (soil nitrogen and phosphorus pools). We calculated the aboveground and belowground biomass of trees based on allometric equations (Table S1). EMF was quantified using the averaging approach. In the averaging approach, the eight functional variables were standardized using Z-score transformation and then averaged to generate an EMF index, which was subsequently used to compare EMF among vegetation types.

### 4.6. Statistical Analysis

To clarify the relationships between EMF and explanatory variables across spatial and temporal gradients, plots along the elevational and successional gradients were analyzed separately using linear models. Spearman correlation analyses were conducted on the explanatory variables for forests along each gradient, indicating no significant collinearity (Figures S1 and S2). Variance partitioning analysis was then used to quantify the contributions of different factor groups to EMF variation. These groups included climate (mean annual temperature, mean annual precipitation), soil (bulk density, soil water content, soil porosity, pH), biodiversity (species richness, functional diversity), community-level functional traits (Hmax, SLA, wood density, leaf thickness), and stand structural attributes (coefficients of variation in DBH and tree height). Negative variance values were interpreted as zero, indicating that the variation explained by the factor group was smaller than expected from random normal variables. Partial least squares path modeling (PLS-PM) was based on the selected variables to elucidate how factors influence EMF. Partial correlation analyses were conducted to confirm the robustness of the identified significant variables. Multiple complementary approaches were employed to robustly identify indicators exerting strong influences on EMF variation. Generalized additive models were applied to examine changes in complementarity and selection effects, with effect sizes represented by the slopes of EMF versus community-weighted mean (CWM) or functional dispersion (FDis) derived from linear models. All statistical analyses were performed using R version 3.5.1 (R Core Team, Vienna, Austria, 2018).

## 5. Conclusions

At spatiotemporal scales in subalpine forests, plant species richness had no significant effect on ecosystem multifunctionality (EMF), challenging many previously assumed positive relationships and highlighting strong regional variability in biodiversity–ecosystem functioning (BEF) patterns. By contrast, plant functional diversity exerted a consistently positive influence on EMF. In high-elevation forests, complementarity effects were significantly stronger than selection effects, indicating that BEF relationships are amplified under harsh climatic conditions. During late successional stages, complementarity effects remained higher than selection effects, although the difference was not statistically significant. Structural diversity, particularly the coefficient of variation in DBH, emerged as an important positive regulator of EMF, underscoring the need to consider vertical and horizontal forest structure in management practices. Among soil properties, sites characterized by high water content and low bulk density were most favorable for enhancing EMF. Collectively, these findings provide mechanistic insights and practical guidance for promoting EMF in subalpine forests and inform the management and restoration of natural forest ecosystems.

## Data Availability

Data will be made available upon request.

## Supplementary Materials

The following supporting information can be downloaded at https://www.mdpi.com/article/10.3390/plants15030518/s1, Table S1. Presents the biomass equations for the main dominant tree species in subalpine forests of Sichuan Province, Figure S1. Correlations among biotic and environmental factors influencing ecosystem multifunctionality (EMF) along the elevational gradient in subalpine forests, Figure S2. Correlations among biotic and environmental factors influencing ecosystem multifunctionality (EMF) along the successional gradient in subalpine forests, Figure S3. A partial correlation analysis of biotic and environmental factors influencing ecosystem multifunctionality in subalpine forests along the elevational gradient, Figure S4. A partial correlation analysis of biotic and environmental factors influencing ecosystem multifunctionality in subalpine forests along the successional gradient, Figure S5. Effects on ecosystem multifunctionality (EMF) along the elevational gradient in subalpine forests.
