# Supplementary material for: Forest Attributes and Soil Moisture Availability Drive Ecosystem Multifunctionality of Forests in Eastern Tibetan Plateau, China
Source: Plants (Basel). 2026 Feb 6;15(3):518. doi: 10.3390/plants15030518 (PMC12899478; doi:10.3390/plants15030518)
Supplement: Supplementary file 1 [file plants-15-00518-s001.zip › plants-4051229-supplementary.pdf]

Supplementary data

Table S1. presents the biomass equations for the main dominant tree species in subalpine forests of Sichuan Province. The equation $W=a_1(D^2H)^{b2}$ defines W as the biomass of the whole tree or a specific organ (kg), D as diameter at breast height (cm), H as tree height (m), a_1, and a_2 as coefficients associated with wood density, b1 and b2 as coefficients associated with the growth environment.

**Province (Autonomous Region)**	**Tree species and functional groups**	**Component**	**a**	**b**	**r²**
	Quercus spp.	Stem	0.0540	0.9080	0.87
		Branch	0.0040	1.0200	0.80
		Leaf	0.0330	0.5470	0.51
		Root	0.0050	1.0490	0.88
	Pinus massoniana	Stem	0.0360	0.9120	0.98
		Branch	0.0150	0.8160	0.90
		Leaf	0.0260	0.6600	0.82
		Root	0.0520	0.7130	0.65
	Alnus spp.	Stem	0.0630	0.8330	0.98
		Branch	0.0390	0.7010	0.88
Sichuan		Leaf	0.1090	0.4070	0.73
		Root	0.1950	0.3430	0.48
	Cunninghamia lanceolata	Stem	0.0750	0.7770	0.95
		Branch	0.0720	0.5690	0.80
		Leaf	0.1650	0.4380	0.58
		Root	0.0570	0.6230	0.74
	Larix mastersiana	Stem	0.0460	0.8470	0.98
		Branch	0.0470	0.6180	0.70
		Leaf	0.0310	0.5660	0.81
		Root	0.0140	0.8200	0.92
	Cinnamomum camphora	Stem	0.0260	0.9590	0.99
		Branch	0.0160	0.8480	0.81
		Leaf	0.0190	0.7050	0.83

		Root	0.0050	1.0150	0.97
	Populus spp.	Stem	0.2380	0.6870	0.96
		Branch	0.0420	0.7710	0.89
		Leaf	0.1310	0.4310	0.58
		Root	0.1150	0.6270	0.85
	Phoebe spp.	Stem	0.0280	0.9490	0.96
		Branch	0.0420	0.7710	0.89
Sichuan		Leaf	0.1310	0.4310	0.58
		Root	0.1150	0.6270	0.85
	Schima spp.	Stem	0.0170	1.0330	0.98
		Branch	0.0030	0.9980	0.92
		Leaf	0.0220	0.6700	0.92
		Root	0.0030	1.0370	0.97
	Pinus yunnanensis	Stem	0.1410	0.6950	0.74
		Branch	0.340	0.7160	0.65
		Leaf	0.0570	0.5700	0.72
		Root	0.0720	0.5810	0.59
	Picea spp.	Stem	0.0730	0.8460	0.91
		Branch	0.0669	0.7160	0.70
		Leaf	0.0420	0.6850	0.69
		Root	0.0340	0.7990	0.87

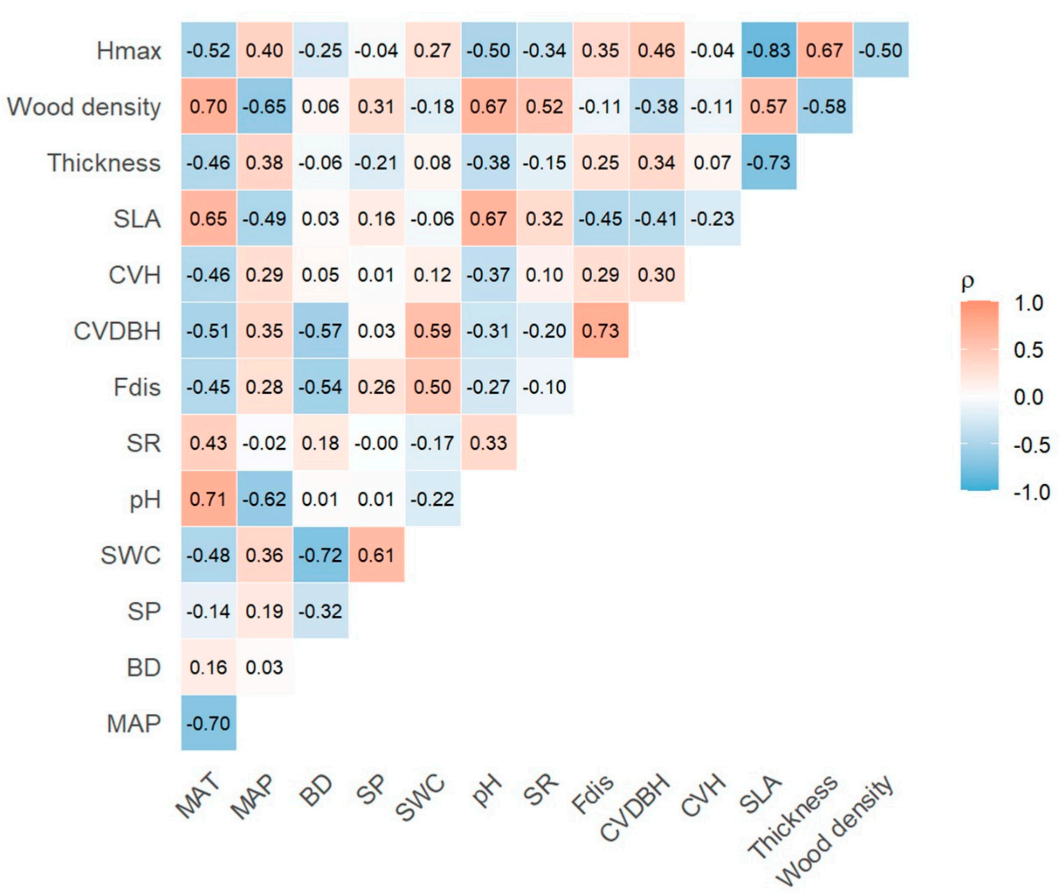

Figure S1. Correlations among biotic and environmental factors influencing ecosystem multifunctionality (EMF) along the elevational gradient in subalpine forests. Factors include soil bulk density (BD), soil water content (SWC), pH, soil porosity (SP), functional diversity (Fdis), species richness (SR), specific leaf area (SLA), leaf thickness, wood density, community maximum tree height (Hmax), coefficients of variation of DBH (CVDBH) and tree height (CVH), mean annual temperature (MAT), and mean annual precipitation (MAP).

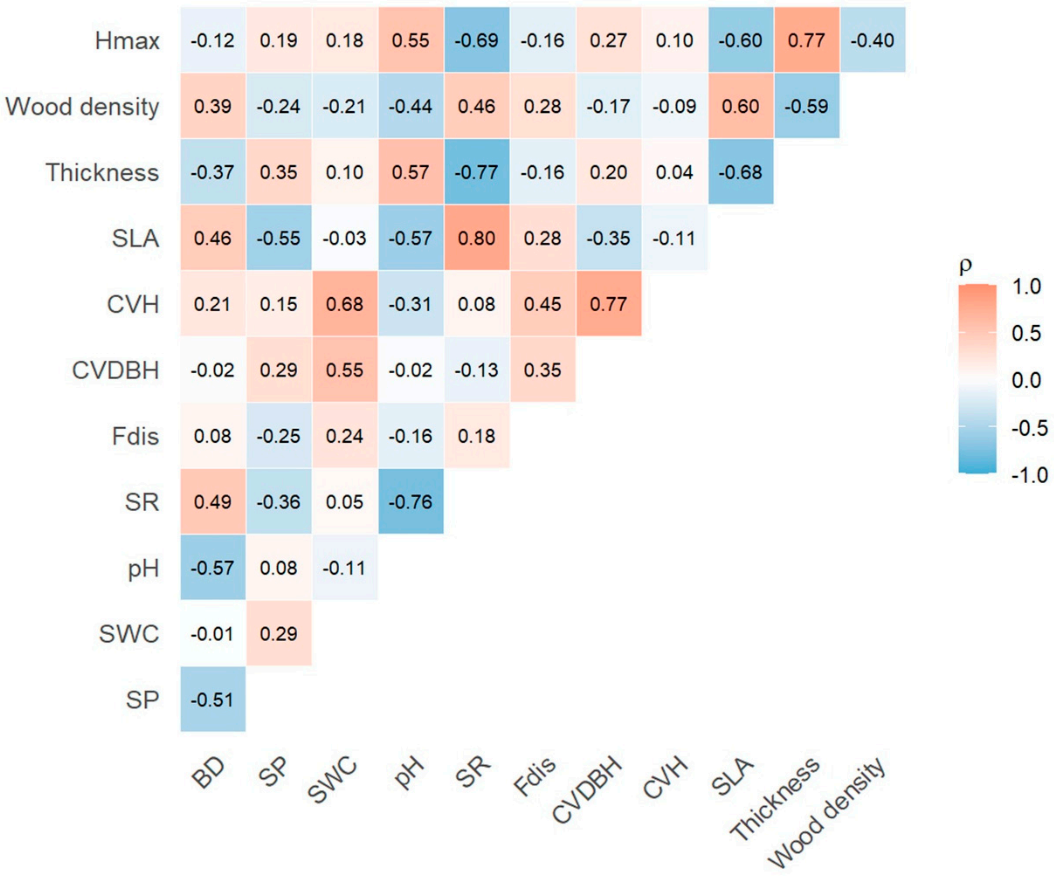

Figure S2. Correlations among biotic and environmental factors influencing ecosystem multifunctionality (EMF) along the successional gradient in subalpine forests. Factors include soil bulk density (BD), soil water content (SWC), pH, soil porosity (SP), functional diversity (Fdis), species richness (SR), specific leaf area (SLA), leaf thickness, wood density, community maximum tree height (Hmax), coefficients of variation of DBH (CVDBH) and tree height (CVH).

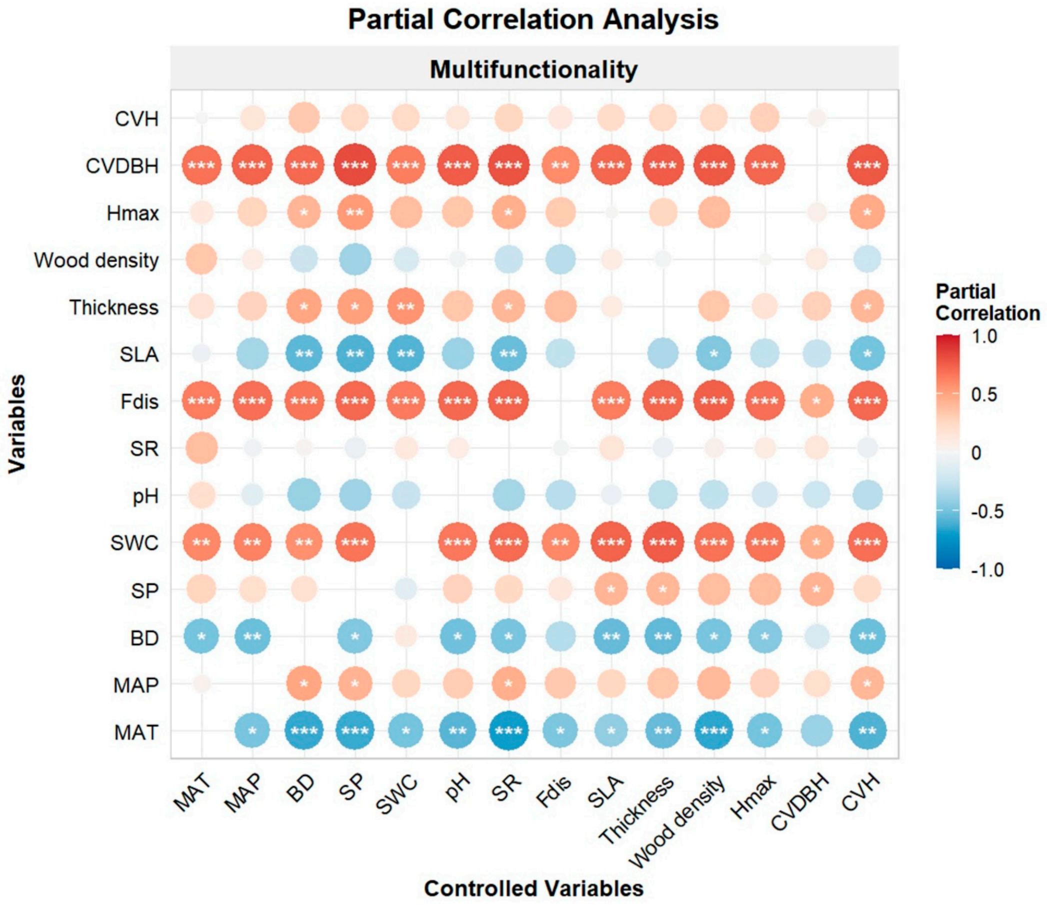

Figure S3. A partial correlation analysis of biotic and environmental factors influencing ecosystem multifunctionality in subalpine forests along the elevational gradient. The analyzed factors include soil bulk density (BD), soil water content (SWC), pH, soil porosity (SP), functional diversity (Fdis), species richness (SR), specific leaf area (SLA), leaf thickness, wood density, community maximum tree height (Hmax), coefficients of variation of DBH (CVDBH) and tree height (CVH), mean an-nual temperature (MAT), and mean annual precipitation (MAP). The color intensity represents the magnitude of partial correlation coefficients, with *P < 0.05, **P < 0.01, and ***P < 0.001.

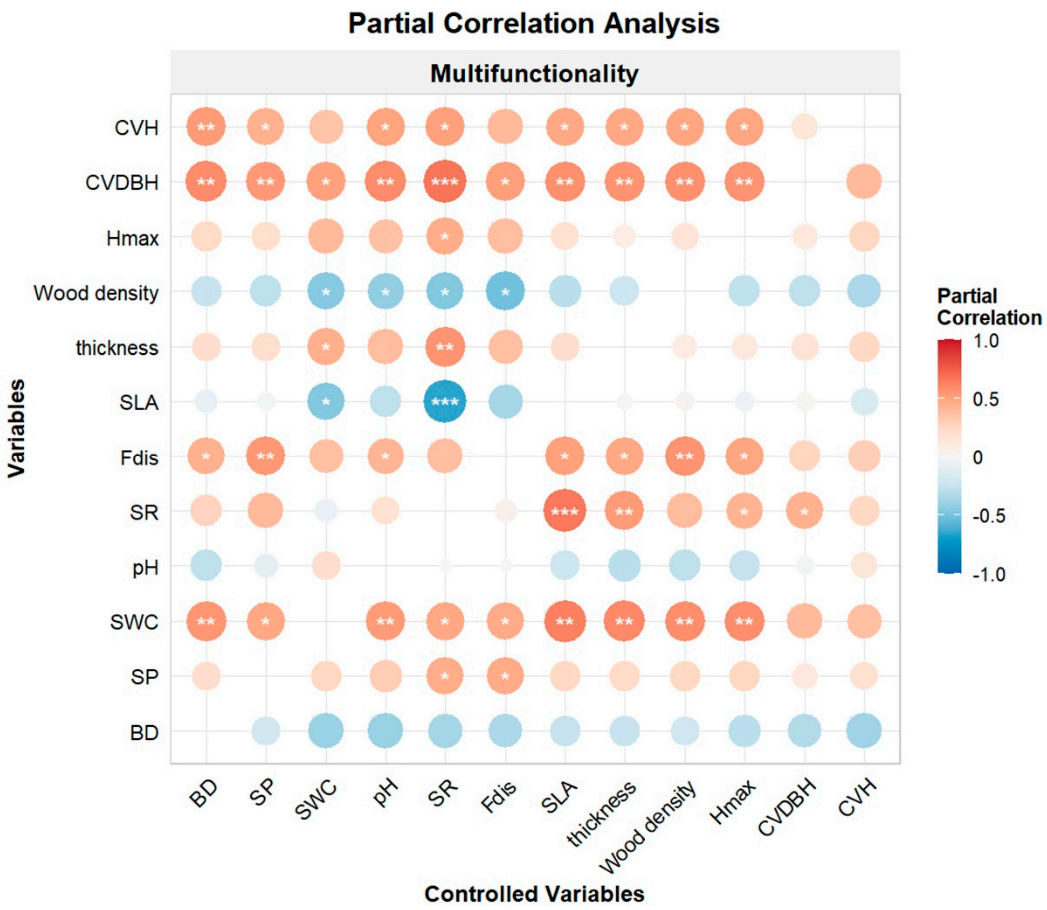

Figure S4. A partial correlation analysis of biotic and environmental factors influencing ecosystem multifunctionality in subalpine forests along the successional gradient. The analyzed factors include soil bulk density (BD), soil water content (SWC), pH, soil porosity (SP), functional diversity (Fdis), species richness (SR), specific leaf area (SLA), leaf thickness, wood density, community maximum tree height (Hmax), coefficients of variation of DBH (CVDBH) and tree height (CVH). The color intensity represents the magnitude of partial correlation coefficients, with *P < 0.05, **P < 0.01, and ***P < 0.001.

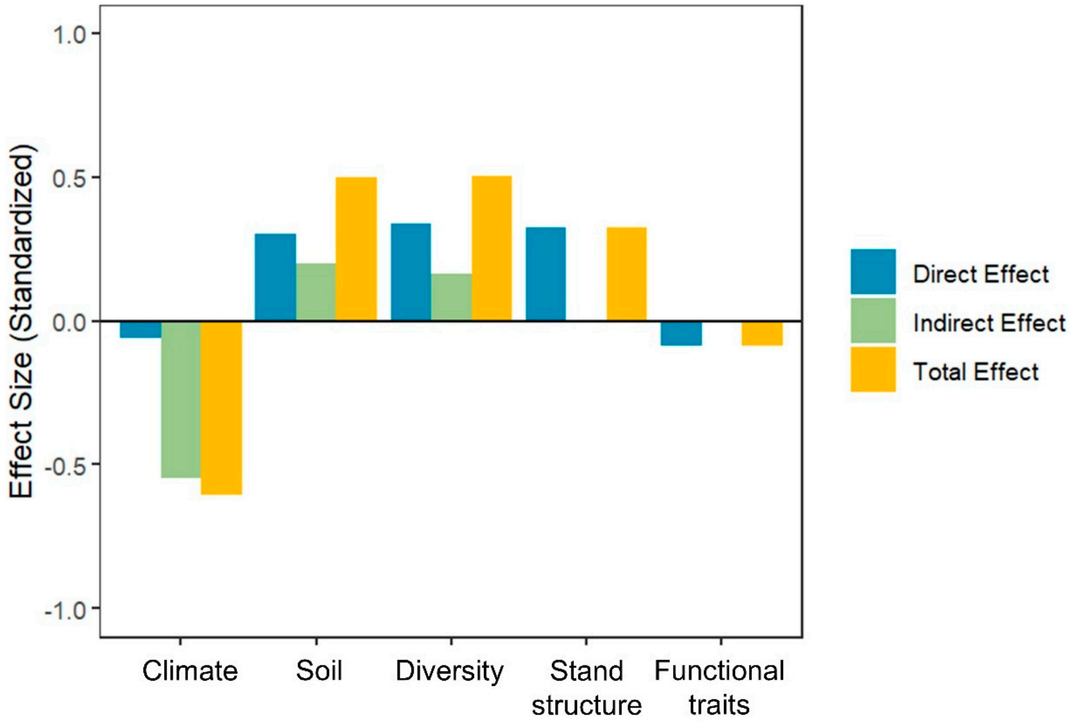

Figure S5. Effects on ecosystem multifunctionality (EMF) along the elevational gradient in subalpine forests.
